# Identification of the Genetic Requirements for Zinc Tolerance and Toxicity in *Saccharomyces cerevisiae*

**DOI:** 10.1534/g3.119.400933

**Published:** 2019-12-13

**Authors:** Yun-ying Zhao, Chun-lei Cao, Ying-li Liu, Jing Wang, Jie Li, Shi-yun Li, Yu Deng

**Affiliations:** * National Engineering Laboratory for Cereal Fermentation Technology (NELCF), School of Biotechnology, Jiangnan University, 1800 Lihu Road, Wuxi, Jiangsu 214122, China,; † China-Canada Joint Lab of Food Nutrition and Health (Beijing), Beijing Technology & Business University, Beijing 100048, China, and; ‡ School of Biotechnology, Jiangnan University, 1800 Lihu Road, Wuxi, Jiangsu 214122, China

**Keywords:** *Saccharomyces cerevisiae*, zinc toxicity, genetic screening, genomics, Reactive oxygen species (ROS)

## Abstract

Zinc is essential for almost all living organisms, since it serves as a crucial cofactor for transcription factors and enzymes. However, it is toxic to cell growth when present in excess. The present work aims to investigate the toxicity mechanisms induced by zinc stress in yeast cells. To this end, 108 yeast single-gene deletion mutants were identified sensitive to 6 mM ZnCl_2_ through a genome-wide screen. These genes were predominantly related to the biological processes of vacuolar acidification and transport, polyphosphate metabolic process, cytosolic transport, the process utilizing autophagic mechanism. A result from the measurement of intracellular zinc content showed that 64 mutants accumulated higher intracellular zinc under zinc stress than the wild-type cells. We further measured the intracellular ROS (reactive oxygen species) levels of 108 zinc-sensitive mutants treated with 3 mM ZnCl_2_. We showed that the intracellular ROS levels in 51 mutants were increased by high zinc stress, suggesting their possible involvement in regulating ROS homeostasis in response to high zinc. The results also revealed that excess zinc could generate oxidative damage and then activate the expression of several antioxidant defenses genes. Taken together, the data obtained indicated that excess zinc toxicity might be mainly due to the high intracellular zinc levels and ROS levels induced by zinc stress in yeast cells. Our current findings would provide a basis to understand the molecular mechanisms of zinc toxicity in yeast cells.

The transition metal ion, zinc, is an essential cofactor for transcription factors and enzymes in all eukaryotic cells as well as an essential nutrient for life in all living organisms (Hambidge and Krebs 2007). However, it is toxic to cell growth when present in excess by generating reactive hydroxyl radicals and disturbing the cellular redox potential (Singh *et al.* 2017). In addition, excess zinc competes for the binding sites in functional proteins for other metals (King *et al.* 2000). Although Zinc can function as a member of antioxidant properties, it generates reactive oxygen species when yeast cells were exposed to high zinc levels (Pagani *et al.* 2007; Powell 2000; Hao and Maret 2005). Moreover, metal toxicities are often attributed mainly to the capacity to induce the unfolded protein response (UPR), the oxidative stress, DNA damage or even cell death (Nargund *et al.* 2008; Muthukumar and Nachiappan 2010; Liu *et al.* 2018). High intracellular ROS levels induced by zinc or other metals and stresses can trigger several biological molecules, such as DNA damage, lipid peroxidation and depletion of protein sulphydryl (Howlett and Avery 1997; Chrestensen *et al.* 2000; Serero *et al.* 2008). Therefore, the intracellular zinc levels must be tightly regulated to maintain zinc homeostasis in an optimal level regardless of its supply. As a model organism, the budding yeast *Saccharomyces cerevisiae* is used to study the basic mechanisms of many cellular processes, including zinc transport and zinc homeostasis (Wu *et al.* 2008).

In budding yeast, zinc homeostasis is tightly sustained via various transporters. Yeast cells assimilate the extracellular zinc through the high and low-affinity transport at the plasma membrane (Eide 2009). Cells uptake the extracellular zinc efficiently via a high-affinity zinc transporter Zrt1, and two low-affinity zinc transports, Zrt2 and Fet4, which are all regulated by the transcriptional factor Zap1 (Waters and Eide 2002; Zhao and Eide 1996a, 1996b). Inside the cell, two vacuolar zinc transporters Zrt3 and Zrc1 are responsible for transporting zinc out or into the vacuolar, respectively, and the heteromeric complex formed by Msc2 and Zrg17 transports the cytoplasm zinc to the endoplasmic reticulum when it is in excess. Interestingly, the three transporters Zrt3, Zrc1 and Zrg17 are also regulated by Zap1 in response to zinc level (MacDiarmid *et al.* 2000; Miyabe *et al.* 2000; Wu *et al.* 2011). The Fet4 transporter involved in uptake of iron and copper and the high-affinity phosphate transporter Pho84 can also uptake zinc (Waters and Eide 2002; Bun-Ya *et al.* 1991).

Zap1 was the first identified fungal zinc-responsive transcription factor from *S. cerevisiae* (Zhao and Eide 1997). Zap1 regulates the expression of about 80 genes by binding to their ZREs (Zinc Responsive Elements) in the promoter regions, including the genes required for zinc homeostasis or survival for a long period of zinc starvation (Zhao and Eide 1997; De Nicola *et al.* 2007; North *et al.* 2012; Zhao *et al.* 1998). At the transcriptional level, Zap1 autoregulates its coding gene *ZAP1* by binding to a ZRE within its own promoter (Zhao *et al.* 1998). Zap1 contains seven C2H2-type zinc fingers. Five of these zinc fingers are needed for DNA binding, while the other two are involved in zinc sensing to regulate AD2 (Wilson and Bird 2016). For the post-translational/ regulation of Zap1, zinc regulates the activities of the Zap1 DNA binding domain, AD1 and AD2 independently (Bird *et al.* 2003; Herbig *et al.* 2005; Frey *et al.* 2011; Zhao *et al.* 1998). Hence, the activity of Zap1 is strongly enhanced in response to zinc limitation, inducing the expression of *ZRT1* encoding a high-affinity zinc transporter while inhibiting the expression of *ZRT2* encoding a low-affinity zinc transporter (Eide 2003; Wilson and Bird 2016). When espoused to high extracellular zinc concentrations, Zrt1 is removed from the plasma membrane rapidly by substrate-induced endocytosis (Schothorst *et al.* 2017; Gitan and Eide 2000; Gitan *et al.* 1998). The expression level of *ZRT1* is induced over 100-fold in zinc-limited cells by Zap1, while *ZRT2* is induced under mild zinc limitation but then inhibited by Zap1 in severe zinc limitation conditions (Zhao and Eide 1997; Bird *et al.* 2004).

Despite these discoveries, it is still largely unknown about the basic mechanisms by which proteins or pathways regulate the cytosolic zinc homeostasis or zinc toxicity. Using yeast as a model system, it is easy to know how a single-gene mutant regulates zinc homeostasis in response to high zinc (Bleackley *et al.* 2011). The present work aims to investigate the toxicity mechanisms induced by zinc stress in yeast cells. We first screened the zinc-sensitive mutants from a collection of *S. cerevisiae* deletion mutants. Specifically, the impact of high intracellular zinc concentration and ROS production were both addressed in this work. Also, we analyzed the expression of genes involved in antioxidant defenses in response to excess zinc.

## Materials and Methods

### Yeast strains and growth conditions

Diploid strains used in this work derived from the BY4743 genetic background. Yeast cells were grown at 30° in YPD medium (1% yeast extract, 2% peptone, 2% glucose). ZnCl_2_ was purchased from Sangon Biotech (Shanghai, China), and dihydroethidium was purchased from Sigma (Beijing, China).

### Genome-wide screen for zinc-sensitive mutations

A collection of *S. cerevisiae* deletion mutants of 4,757 non-essential genes in BY4743 background were purchased from Thermo Fisher SCIENTIFIC (http://clones.thermofisher.com/cloneinfo.php?clone=yeast, catalog number: 95400.BY4743) and frozen at -80° in 96-well microtitre plates in liquid YPD medium containing 15% glycerol. The primary screen of zinc-sensitive mutations was first performed by transferring the deletion mutant library to fresh liquid YPD medium (pH ∼5.5) and cultured at 30° in new 96-well microtitre plates. Then, 20 μL of each mutant was transferred to 180 μL fresh liquid YPD medium with or without 3 mΜ ZnCl_2_ and incubated at 30° for 12 h. Mutants with a relative OD_600_ reduced by more than 30% in liquid YPD medium supplemented with ZnCl_2_ relative to wild-type cells but not in liquid YPD medium were considered zinc-sensitive. Mutants that appeared sensitive were confirmed by the serial dilution assay method in solid YPD plates, YPD plates with 6 mΜ ZnCl_2_, YPD plates with 6 mΜ ZnSO_4_ and YPD plates with 12 mΜ NaCl as described previously (Zhao *et al.* 2013). Namely, the individual deletion mutants grown overnight in liquid YPD at 30° and serially diluted by 10 times with ddH_2_O. Each dilution of 2.5 µL was spotted onto the above indicated plates and incubated at 30° for 2-3 days.

### Determination of the intracellular zinc concentrations

To determinate of the total intracellular zinc concentrations in the 108 zinc-sensitive mutants, cells were first cultured in YPD media to middle log phase and then were treated with 3 mM ZnCl_2_ for 2 hr. Next, cells were collected and prepared for measuring the intracellular zinc concentration using an atomic absorption spectrometer in flame emission mode as described previously (Zhao *et al.* 2013). Three individual colonies for each mutant were measured, where the wild-type BY4743 cells served as a control.

### Detection of the intracellular ROS accumulation

The intracellular ROS level was monitored with dihydroethidium (DHE) (Büttner *et al.* 2007). Before zinc treatment, cells were cultured in YPD media to the middle log phase and split into two aliquots. Subsequently, yeast cells were exposed to YPD supplemented with or without 3 mM ZnCl_2_ or 8 mM ZnCl_2_ for 2 hr or 5 hr, respectively. The relative fluorescence units (RFU) were measured in a Synergy H4 fluorescence reader (BioTek) at a fluorescence excitation of 485nm and an emission of 535nm. The RFU was corrected and normalized by the density of OD_600_ of each corresponding mutant. Three individual colonies for each mutant were measured, where wild-type BY4743 cells served as a control.

### RNA extraction and quantitative real-time PCR (qPCR) assay

To extract the total RNA, yeast cells were first grown in YPD to a density of OD_600_ = 0.6∼0.8 and were spilt into aliquots Then cells were cultured in YPD supplemented with or without 3 mM ZnCl_2_ for an additional 1 hr. Cultures were then collected and the total RNA was extracted by the hot phenol method. The first-strand cDNA of each sample was synthesized via the Primer Script RT reagent kit (Cwbiotech, China) following the manufacturer’s instructions. Quantitative PCR (qPCR) method was used to detect the relative expression levels of *TRR1*, *TRX2*, *SOD1*, *GSH1*, *CTT1*, *GPX2* with primer pairs listed in Table S1, respectively. The *PGK1* gene was used as an internal control. qPCR reactions were performed in a Thermo Scientific CFX96 instrument using SYBR Premix Ex Taq (Cwbiotech, China). The results were abstained through the –ΔΔCt method (Livak and Schmittgen 2001). Each reaction was carried out in triplicate.

### Meta-analysis and protein-protein interaction (PPI) network construction for the identified genes

Gene Ontology (GO) enrichment analyses of the zinc-sensitive genes were performed using the powerful web-based Metascape tool (http://metascape.org/gp/index.html#/main/step1). P-value < 0.01 was set as the cutoff criterion, and significance was ranked by enrichment score (− log 10 (P-value)). A web-based search tool, STRING (http://string-db.org), was selected to explore the potential protein-protein interactions of the screened zinc-sensitive genes. A reliability threshold of a combined score of >0.4 was considered as significant interaction pair and then protein-protein interaction (PPI) network was constructed and visualized by Cytoscape software (version 3.6.0, http://www.cytoscape.org/) (Shannon *et al.* 2003).

### Data availability

Strains are available upon request. The authors affirm that all data necessary for confirming the conclusions of the article are present within the article, figures, and tables. Supplemental material available at figshare: https://doi.org/10.25387/g3.11365043.

## Results

### Identification of genes involved in zinc tolerance

To identify the genes involved in zinc tolerance, we screened the yeast diploid nonessential gene deletion library. The results indicated that a set of mutants for 108 genes showed reduced growth in exposing to 6 mM ZnCl_2_ (Table 1). The zinc sensitivity of 48 mutants has been reported (http://www.yeastgenome.org) (Table 1, underlined; Table S2), while the zinc sensitivity of the other 60 mutants is reported for the first time. The functional classes of these 108 are related to metabolism (11), cell cycle and DNA processing (18), transcription (15), Protein fate (synthesis, folding, modification, destination) (17), cellular transport, transport facilities and transport routes (32), as well as unclassified proteins (15) (Table 1).

**Table 1:** List of 108 genes whose deletion mutants are sensitive to 6 mM ZnCl_2_

Systemic name	Standard name	Systemic name	Standard name	Systemic name	Standard name	Systemic name	Standard name
**Metabolism (11)**							
YDR127W*^a^*	*ARO1*	YHR106W	*TRR2*	YNL129W	*NRK1*	YPR060C	*ARO7*
YGL234W	*ADE5,7*	YJL216C	*IMA5*	YNL220W	*ADE12*	YDL133W*^b^*	*SRF1*
YHR004C	*NEM1*	YLR425W	*TUS1*	YPL268W	*PLC1*		
**Cell cycle and DNA processing (18)**							
YAL015C	*NTG1*	YDL047W	*SIT4*	YJL092W	*SRS2*	YNL271C	*BNI1*
YAL020C	*ATS1*	YDL101C	*DUN1*	YJL208C	*NUC1*	YPL031C	*PHO85*
YAL047C	*SPC72*	YDR137W	*RGP1*	YJR043C	*POL32*	YHR113W	*APE4*
YCR077C	*PAT1*	YER116C	*SLX8*	YLL002W	*RTT109*		
YCR086W	*CSM1*	YHR061C	*GIC1*	YNL059C	*ARP5*		
**Transcription (15)**							
YCR081W	*SRB8*	YFL001W	*DEG1*	YHR178W	*STB5*	YLR182W	*SWI6*
YDL160C	*DHH1*	YFL049W	*SWP82*	YIL154C	*IMP2’*	YNR052C	*POP2*
YDR028C	*REG1*	YGL071W	*AFT1*	YJL124C	*LSM1*	YPL254W	*HFI1*
YDR310C	*SUM1*	YGR092W	*DBF2*	YKL139W	*CTK1*		
**Protein fate (synthesis, folding, modification, destination) (17)**							
YBR131W	*CCZ1*	YDR283C	*GCN2*	YJL004C	*SYS1*	YNL069C	*RPL16B*
YCL001W	*RER1*	YFL016C	*MDJ1*	YJL204C	*RCY1*	YPR133W-A	*TOM5*
YCL045C	*EMC1*	YGL124C	*MON1*	YKL006W	*RPL14A*		
YCR079W	*PTC6*	YGL195W	*GCN1*	YLL010C	*PSR1*		
YDL045W-A	*MRP10*	YHR076W	*PTC7*	YMR264W	*CUE1*		
**Cellular transport, transport facilities and transport routes (32)**							
YBR127C	*VMA2*	YFL004W	*VTC2*	YJL012C	*VTC4*	YLR261C	*VPS63*
YCL038C	*ATG22*	YGL212W	*VAM7*	YJL024C	*APS3*	YLR262C	*YPT6*
YCR037C	*PHO87*	YGL095C	*VPS45*	YJL154C	*VPS35*	YLR268W	*SEC22*
YDL100C	*GET3*	YGR105W	*VMA21*	YJL198W	*PHO90*	YLR396C	*VPS33*
YDR089W	*VTC5*	YHL031C	*GOS1*	YLR242C	*ARV1*	YMR243C	*ZRC1*
YDR186C	*SND1*	YHR026W	*VMA16*	YKL080W	*VMA5*	YNL323W	*LEM3*
YDR276C	*PMP3*	YHR094C	*HXT1*	YKL119C	*VPH2*	YOR270C	*VPH1*
YDR456W	*NHX1*	YHR108W	*GGA2*	YKR020W	*VPS51*	YPL045W	*VPS16*
**Unclassified proteins (15)**							
YCL007C		YHR112C		YKL044W	*MMO1*	YNL204C	*SPS18*
YDL041W		YHR151C	*MTC6*	YLR149C		YPL261C	
YDR203W		YJL211C		YLR232W		YPR123C	
YDR417C		YJL218W		YMR265C			

a Gene names were listed alphabetically according to their systemic names.

b The mutations for 48 genes that were reported sensitive to zinc previously were underlined.

**Figure 1:**
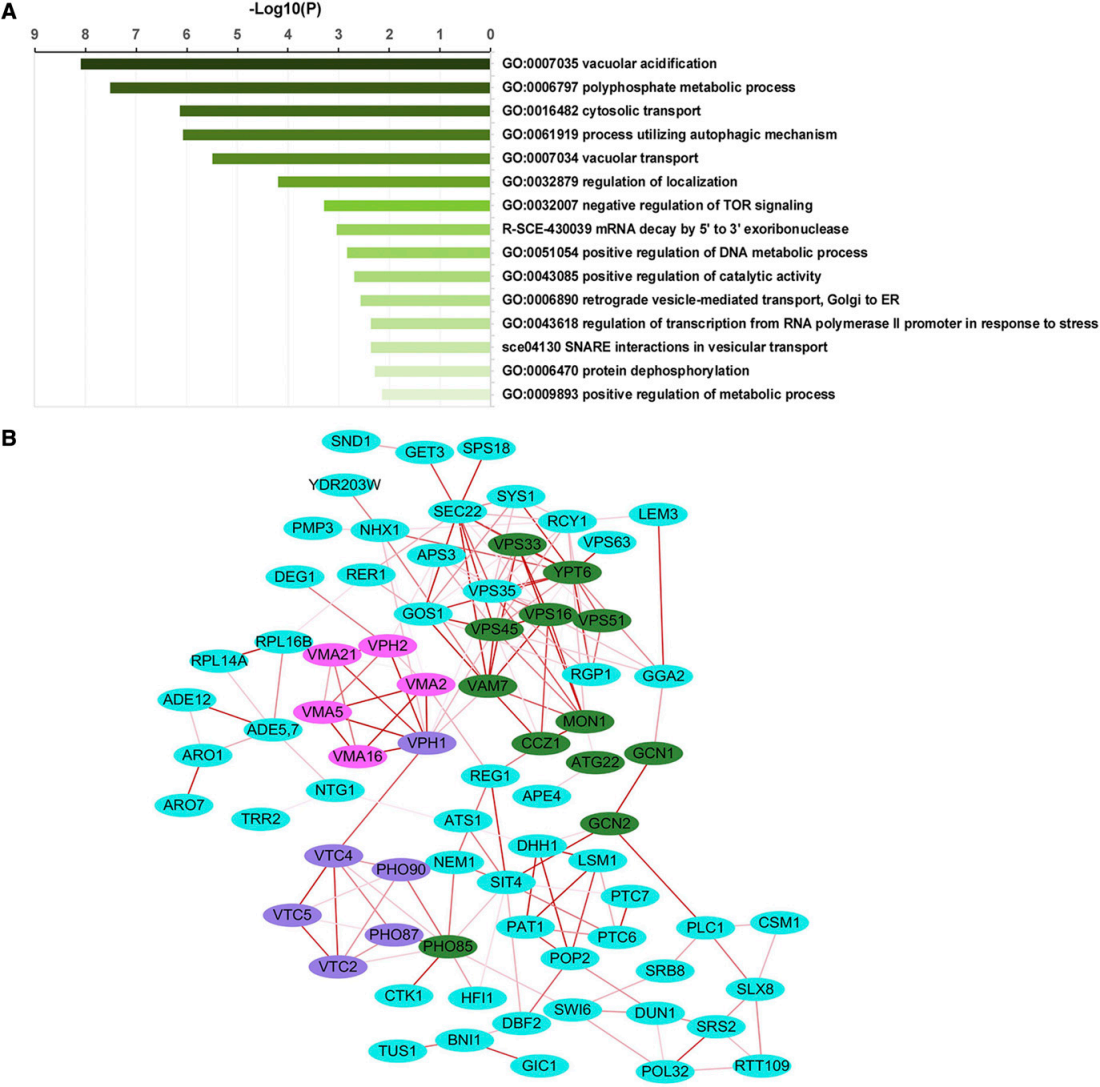
Meta-enrichment analysis summary of zinc-sensitive genes. (A) Heatmap of the top 20 enriched GO terms. For GO terms, each band represents one enriched term colored according to its -log 10 *p*-value. The dominant term within each group is used as a group heading. (B) The protein-protein interaction (PPI) networks of the zinc-sensitive genes. The edges represent the combined score, and the thicker the edge, the higher the similarity.

Gene Ontology (GO) enrichment analysis showed that the 108 genes were most enriched in the biological processes of vacuolar acidification, polyphosphate metabolic process, cytosolic transport, the process utilizing autophagic mechanism and vacuolar transport among the top 20 GO terms in cluster groups (Figure 1A). All the zinc-sensitive genes were then uploaded to the web-based online tool STRING (https://string-db.org/) and the information of PPI was gained and visualized by Cytoscape software (version 3.6.0) (Figure 1B). A total of 76 proteins were filtered from the 108 candidates, and a significant functional network was constructed by these 76 nodes and 168 interaction pairs.

### Elevated intracellular zinc levels evoked by excess zinc

**Figure 2:**
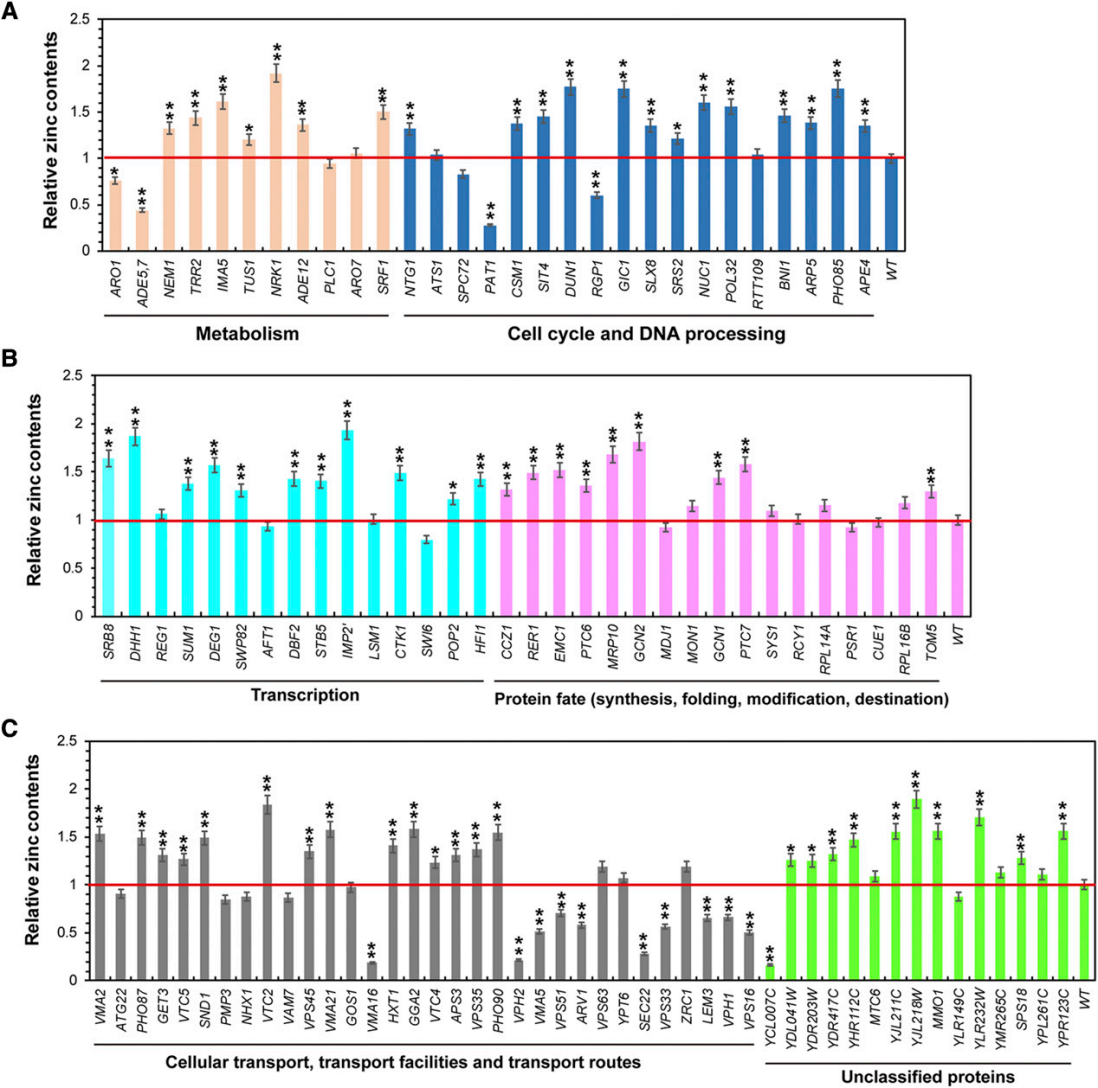
Intracellular zinc levels of 108 zinc-sensitive gene mutants in response to zinc stress. Log-phase cells were grown with or without 3 mM ZnCl_2_ for two hours before they were collected for the measurement of intracellular zinc levels. The intracellular zinc content s of these mutants were listed according to their categories in comparison to that of wild type cell BY4743. The value is the average of three independent assays for each strain. The asterisks of “*”and “**” show statistically significant differences of *P* < 0.05 and *P* < 0.01, respectively.

To determine the correlations between zinc sensitivity and intracellular zinc accumulations in all zinc-sensitive mutants, we analyzed the intracellular zinc concentrations in yeast cells treated with 3 mM ZnCl_2_. We showed that the intracellular zinc content of a number of 64 mutants (almost 59% of the total 108 genes) was significantly increased compared with wild-type cells (Figure 2). Meanwhile, 29 zinc-sensitive mutants accumulated similar intracellular zinc ions compared with the wild-type strain, and mutants for 15 genes (*ARO1*, *ADE5,7*, *PAT1*, *RGP1*, *VMA16*, *VPH1*, *VMA5*, *VPS51*, *ARV1*, *SEC22*, *VPS22*, *LEM3*, *VPH1*, *VPS16* and *YCL007C*) accumulated less intracellular zinc than that of wild-type cells, respectively.

Phosphate homeostasis is regulated by SPX domain proteins in eukaryotes and plays an essential role in the biosynthesis of diverse cellular components (Secco *et al.* 2012). Interestingly, in the present study, deletion mutants for five genes (*PHO87*, *PHO90*, *VTC2*, *VTC4* and *VTC5*) encoding SPX-domain proteins, and one gene (*PHO85*) coding for cyclin-dependent kinase Pho85, were all sensitive to 6 mM ZnCl_2_ and accumulated increased intracellular zinc levels in response to excess zinc (Table 1, Figure 2). Based on these findings, we speculate that phosphate homeostasis is significantly involved in maintaining the intracellular zinc homeostasis under excess zinc conditions in budding yeast.

Yeast V-ATPase, an H^+^-ATPase localized in the vacuole membrane, played crucial roles in regulating the pH values, acidifying intracellular organelles and cellular homeostasis (Graham *et al.* 2000; Couoh-Cardel *et al.* 2016). In the present study, we showed that deletion of the 8 genes related to the regulation of the intracellular pH (*NHX1*, *VMA2*, *VMA21*, *DBF2*, *VPH2*, *VMA5*, *VPH1* and *VMA16*), rendered yeast cells sensitive to 6 mM ZnCl_2_, although only mutants for two genes (*VMA2* and *VMA21*) accumulated higher intracellular zinc in response to zinc stress (Figure 2C). In addition, mutants for 8 genes (*VAM7*, *VPS45*, *APS3*, *VPS35*, *VPS63*, *VPS33* and *VPS51*) encoding proteins associated with the function of vacuolar protein sorting were also sensitive to zinc stress. However, only mutants for *VPS45*, *APS3* and *VPS35* accumulated higher intracellular zinc in response to zinc stress (Figure 2C).

Autophagy is a fundamental cellular process of all eukaryotic cells that aged and/or damaged cytoplasmic proteins, lipids, unwanted organelles and cytosol are translocated to the vacuole and degraded (Song and Kumar 2012; Yin *et al.* 2016). Interestingly, 18 mutants for *ATG22*, *PTC6*, *VTC2*, *YPT6*, *VPS33*, *GCN2*, *CCZ1*, *VAM7*, *GCN1*, *MON1*, *VTC4*, *RPL14A*, *VPS51*, *PHO85*, *VPS16*, *SEC22*, *GOS1* and *VPS45* enriched in the process utilizing autophagic mechanism, were sensitive to zinc (Table 1). Of these genes, mutants for 8 genes (*PTC6*, *VTC2*, *GCN2*, *CCZ1*, *GCN1*, *VTC4*, *PHO85* and *VPS45*) accumulated higher intracellular zinc than wide-type cells, indicating their important roles in maintaining the intracellular zinc homeostasis. The only identified gene ATG gene in this study, *ATG22*, encodes a protein involved in transporting small molecules such as amino acids back to the cytosol for protein synthesis and other cellular functions (Tyler and Johnson 2018). Three genes of *CCZ1*, *MON1* and *YPT6* were required for the CVT pathway and the autophagy (Cabrera *et al.* 2014; Suda *et al.* 2013). VTC (Vacuolar Transporter Chaperone) complex consists of five subunits (Vtc1, Vtc2, Vtc3, Vtc4 and Vtc5), and functions in several membrane-related processes, including the microautophagic scission of vesicles into the vacuolar lumen. Three VTC genes (*VTC2*, *VTC4* and *VTC5*) screened out in the present study and cyclin-dependent kinase gene *PHO85* were all involved in phosphate homeostasis, while Pho85 was also a regulator of autophagy (Reggiori and Klionsky 2013). Gcn2 kinase and its positive regulator Gcn1 were both identified sensitive to high zinc. Gcn2 is involved in sensing the level of intracellular amino acids and can be regulated by transcription factor Gcn4 and the cyclin-dependent kinase Pho85 (Reggiori and Klionsky 2013).

Notably, consistent with a previous study (Pagani *et al.* 2007; Mesquita *et al.* 2016), we found that deletion of *AFT1* encoding a transcription factor, *ZRC1* encoding a protein transporting zinc into vacuolar, rendered yeast cells sensitive to 3 mΜ ZnCl_2_, although the intracellular zinc content of the two mutants reveal no significant differences in response to zinc stress (Figure 2B, C).

### Oxidative stress induced by zinc stress

**Figure 3:**
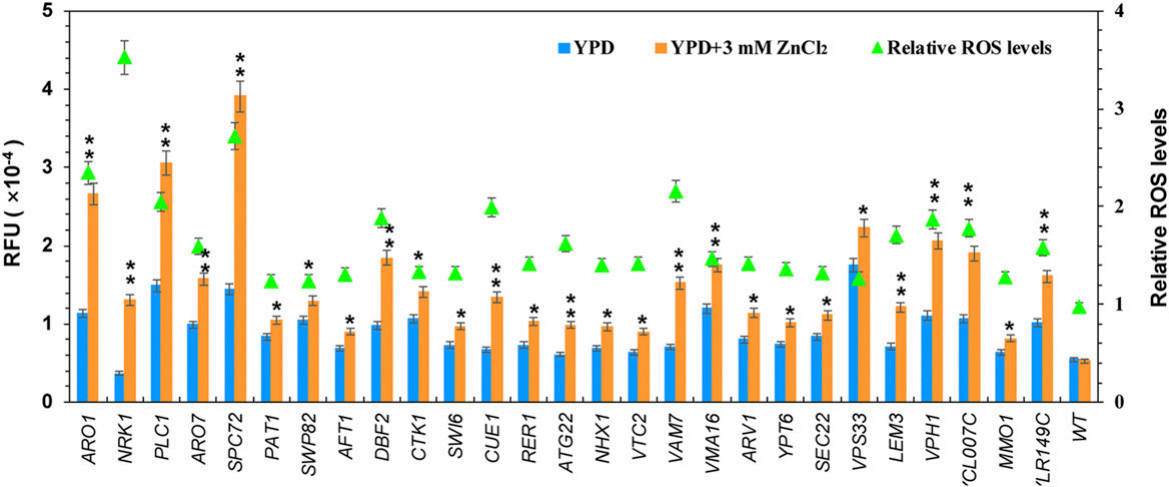
Increased intracellular ROS levels of zinc-sensitive gene mutants in response to 3 mM ZnCl_2_. The relative ROS levels of these mutants in response to zinc treatment are normalized against their related untreated cells. Log-phase cells were grown with or without 3 mM ZnCl_2_ for 2 hr before harvesting and measurement of intracellular ROS levels using dihydroethidium. Results are averages of three independent assays for each strain. The asterisks of “*”and “**” show statistically significant differences of *P* < 0.05 and *P* < 0.01, respectively.

Excess zinc can generate reactive oxygen species (ROS) when it accumulates to toxic levels in cells (Pagani *et al.* 2007). It was reported that ROS stress was induced when the wide-type cells were treated with 10 mM. A low concentration of zinc (5 mg/L, about 77 µM) did not induce ROS production (Mesquita *et al.* 2016), while 5 mM zinc produced a weak ROS response in wild-type cells (Pagani *et al.* 2007). However, some zinc-sensitive genes might be involved in this process. To further confirm this possibility, we measured the intracellular ROS levels following 3 mM ZnCl_2_ or 8 mM ZnCl_2_ treatment, respectively. When the concentration of ZnCl_2_ is 3 mM, the intracellular ROS level in wild-type BY4743 cells treated with zinc showed no significant difference compared with the untreated cells. Of these 108 zinc-sensitive mutants, the intracellular ROS levels of about 27 mutants were increased by extracellular zinc stress (Figure 3), including 11 genes involved in cellular transport (*ATG22*, *NHX1*, *VTC2*, *VAM7*, *VMA16*, *ARV1*, *YPT6*, *SEC22*, *VPS33*, *LEM3* and *VPH1*), five genes of transcriptional process (*SWP82*, *AFT1*, *DBF2*, *CTK1* and *SWI6*), four genes of metabolism (*ARO1*, *NRK1*, *PLC1* and *ARO7*), two gens of cell cycle and DNA processing (*SPC72* and *PAT1*), one genes of protein fate (*CUE1*), and other three unidentified genes (*YCL007C*, *MMO1* and *YLR149C*). It indicated that these 27 genes were all crucial for dealing with the oxidative damage induced by 3 mM ZnCl_2_. The other 81 mutants accumulated similar (73 mutants) or even lower (18 mutants) intracellular ROS levels when treated with zinc compared with the no treated cells (Figure S1), respectively. When these mutants were treated with 8 mM ZnCl_2_, it was showed that 51 mutants displayed increased ROS levels, including 23 mutants in which the intracellular Zn levels were higher than that of the wild type cells (Figure S3). These results suggested that these genes might be involved in the regulation of intracellular ROS levels induced by higher level of zinc.

### The expression of genes involved in redox homeostasis is regulated by zinc stress

**Figure 4:**
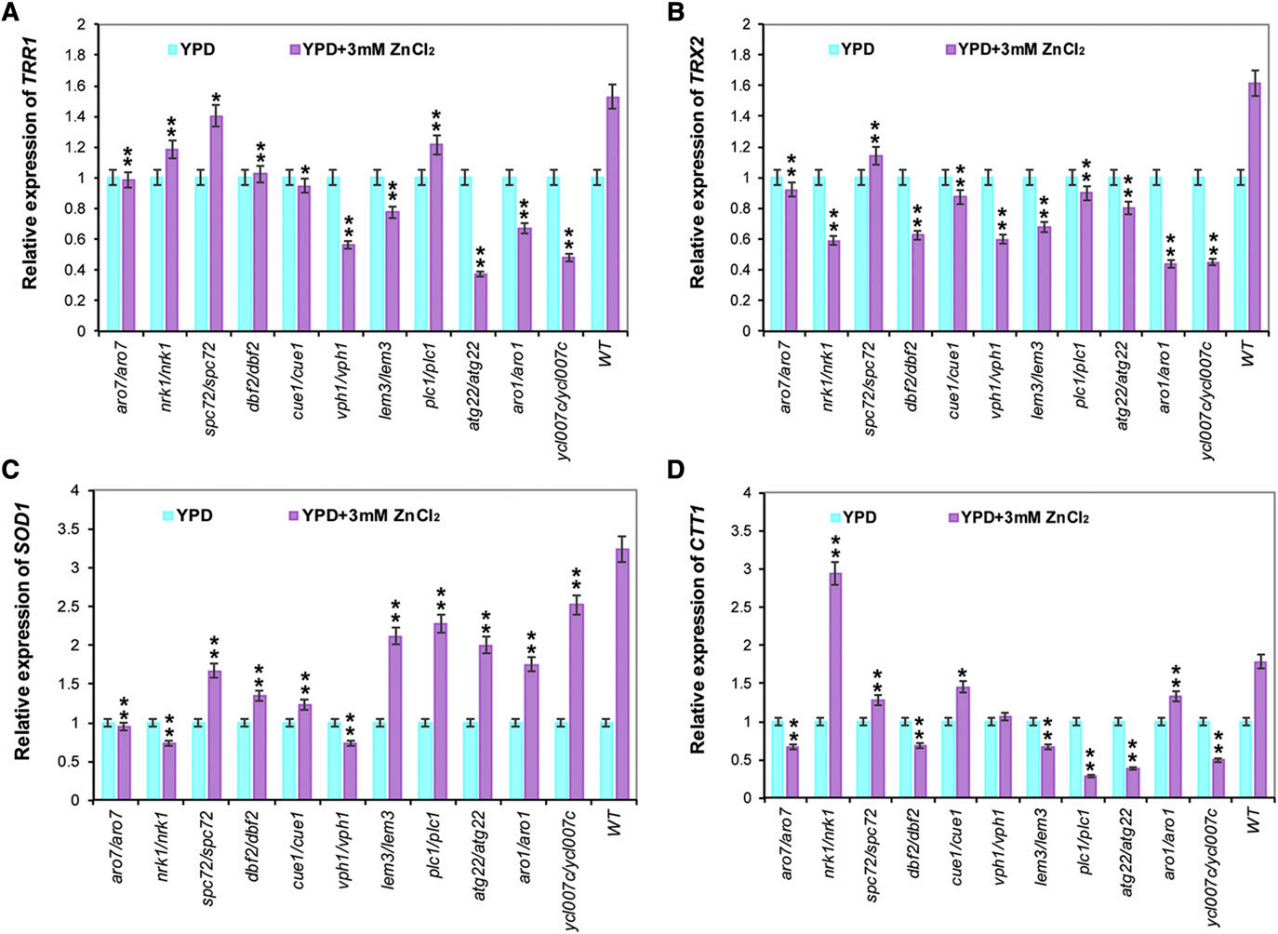
Relative expression levels of genes involved in oxidative stress response. *TRR1*, *TRX2*, *SOD1*, *CTT1*, genes in the *S. cerevisiae*. Gene expression is quantified using RT-qPCR and comparative critical threshold (2 -ΔΔCt) method. The *PGK1* gene was used as internal control and the ratio of the fold-change without treatment was standardized to 1.0. These values represent the average of three independent experiments. The asterisks of “*”and “**” show statistically significant differences of *P* < 0.05 and *P* < 0.01, respectively.

It was reported that zinc induced the expression of a number oxidative stress scavenging genes, including *CTT1*, *GAD1*, *GPX1*, *SOD1* and *SOD2*, etc (Pagani *et al.* 2007). To investigate whether the zinc-sensitive genes were involved in regulating the expression level of genes involved in redox homeostasis, we pick up 11 mutants for *ARO7*, *NRK1*, *SPC72*, *DBF2*, *CUE1*, *VPH1*, *LEM3*, *PLC1*, *ATG22*, *ARO1*, and *YCL007C* accumulated the most relative ROS levels when treatment with zinc (Figure 3), to test the mechanism of oxidative damage induced by zinc. Next, we measured the expression of *TRR1* (thioredoxin reductase), *TRX2* (thioredoxin 2), *GSH1* (glutamylcysteine synthetase), *SOD1* (copper/zinc superoxide dismutase), *CTT1* (cytosolic catalase T) and *GPX2* (2-Cys peroxiredoxin), in these 11 mutants by qPCR assay. Four genes of *TRR1*, *TRX2*, *SOD1* and *CTT1* were significantly up-regulated after treatment with zinc in the wild-type cells (Figure 4), however, no significant difference was showed for the expression of *GSH1* or *GPX2* in the presence or absence of zinc (Figure S2). Interestingly, both of the expression levels of *TRX2* and *SOD1* were decreased in the 11 mutants compared with wild type cells (Figure 4B, C). Furthermore, the expressions of *TRR1* and *CTT1* were also reduced in these mutants except the mutants for *SPC72* and *NRK1*, respectively (Figure 4A, D). Based on these analyses, we concluded that the decreased expression of *TRR1*, *SOD1*, *CTT1* and *TRX2* might be responsible for the high intracellular ROS levels evoked in these mutants.

## Discussion

The main goals of this study were to identify the genes involved in zinc tolerance, and to investigate the toxicity mechanisms induced by zinc stress in yeast cells. Zinc serves as a crucial structural and catalytic cofactor for many functional proteins such as transcriptional factors containing zinc-finger (s), molecular chaperones, DNA or RNA polymerases, lipid-binding proteins, some metabolic enzymes, etc (Singh *et al.* 2017). However, it is toxic at high intracellular levels as zinc can generate reactive oxygen species and thus trigger several biological molecules damaged to cell growth (Howlett and Avery 1997; Chrestensen *et al.* 2000; Serero *et al.* 2008; Pagani *et al.* 2007). Therefore, zinc homeostasis must be tightly controlled. In the present study, we identified 108 zinc-sensitive gene deletion mutations from a genome-scale screen in budding yeast, including 60 mutants that have not been reported previously. It was reported the vacuole integrity was essential for chloride homeostasis (Jennings and Cui 2008), therefore we pick up some of the mutants to investigate whether they are sensitive to another Zn-containing compound ZnSO_4_, as well as NaCl. However, the results showed that these mutants shared the similar sensitivities in 6 mM of ZnCl_2_, ZnSO_4_ and 12 mM NaCl was not affected the growth of these mutants (Figure S3). The results indicated that the zinc sensitivities of these mutants were not influenced by the chloride homeostasis. Our finding might imply that excess zinc toxicity might be mainly due to the high intracellular zinc levels and/or ROS levels induced by zinc stress in yeast cells, although not all mutants revealed high intracellular zinc levels and ROS levels in response to high zinc.

In budding yeast, ten genes encode SPX domain proteins, which are named after S yg1, P ho81, X PR1 (Secco *et al.* 2012). Of the ten SPX domains proteins, nine have been identified to relate to Pi metabolism, including two plasma membrane Pi importers, Pho87 and Pho90 (Wykoff and O’Shea 2001; Bun-ya *et al.* 1996; Hürlimann *et al.* 2007); a vacuolar Pi exporter, Pho91 (Hürlimann *et al.* 2007); a cyclin-dependent kinase inhibitor Pho81, which regulate the activity of Pho80/Pho85 (Schneider *et al.* 1994); and a glycerophosphocholine phosphodiesterase, Gde1 (Fisher *et al.* 2005). Four SPX domains proteins (Vtc2, Vtc3, Vtc4 and Vtc5) involved in producing polyP, are part of the Vacuole Transporter Chaperone (VTC) complex, which play functional roles in sorting of H^+^-translocating ATPases, endocytosis, ER-Golgi trafficking, vacuole fusion, vacuolar polyphosphate homeostasis and the microautophagic scission of vesicles into the vacuolar lumen (Müller *et al.* 2002; Müller *et al.* 2003; Desfougères *et al.* 2016). The tenth SPX domain protein with unknown function is encoded by *SYG1* and localizes in plasma membrane, which might have a function in exporting Pi (Vaughan *et al.* 2012). Interestingly, the results indicated that six genes involved in the process of phosphate mechanism were sensitive to zinc and accumulated higher intracellular zinc, indicating their crucial roles in maintaining the intracellular zinc homeostasis under excess zinc conditions in budding yeast.

**Figure 5:**
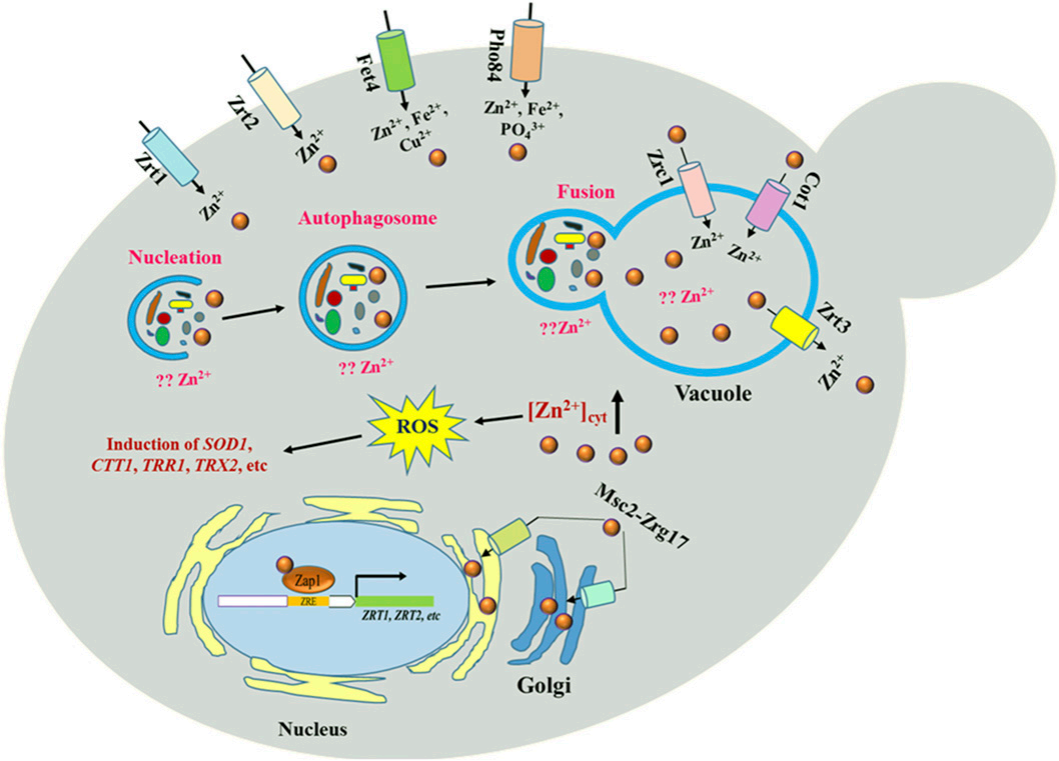
Predicted model for zinc homeostasis and its toxicity mechanisms. Yeast cells uptake the extracellular zinc through plasma membrane transporters Zrt1, Zrt2, Fet4 and Pho84. Inside the cell, two vacuolar zinc transporters Cot1, Zrt3 and Zrc1 are responsible for transporting zinc out or into the vacuolar, respectively, and the heteromeric complex formed by Msc2 and Zrg17 transports the cytoplasm zinc to the endoplasmic reticulum when it is in excess. Excess zinc can generate reactive oxygen species (ROS) induce the oxidative stress scavenging genes. The process of autophagy may play a significant role in maintaining the cellular zinc homeostasis possibly by transporting the excess zinc to vacuolar.

Autophagy is well conserved from budding yeast to human cells and is also crucial for maintaining the cellular and organismal homeostasis, immunity and organismal development (Yin *et al.* 2016). There two types of autophagy in yeast, macroautophagy and microautophagy, a nonspecific or direct process, respectively (Tyler and Johnson 2018). The process of autophagy is regulated by nitrogen, glucose depletion, amino acid and phosphate starvation, mitophagy, pexophagy, transcriptional control and lipid metabolisms (Reggiori and Klionsky 2013). Zinc depletion induces non-selective autophagy in a Zap1-independent manner and this process may play a role in releasing zinc from the degraded proteins for other purposes (Kawamata *et al.* 2017). Interestingly, we identified 18 genes involved in the process of autophagy mechanism, were sensitive to zinc. Two groups of proteins, autophagy-related (ATG) or vacuolar protein sorting (VPS) proteins, mediate the process of autophagy. Importantly, the cytoplasm-to-vacuole targeting pathway (Cvt pathway) is used to deliver various cargos to the vacuole, and is considered a specialized form of autophagy. Furthermore, three genes related to amino acids synthesis (*APE4*, *ARO1* and *ARO7*) and four genes involved in lipid homeostasis (*ARV1*, *SRF1*, *NEM1* and *LEM3*), were all required for zinc tolerance. The SNARE (soluble N-ethylmaleimide-sensitive factor attached protein receptor) complex, a protein complex involved in membrane fusion, is also required for autophagy (Tyler and Johnson 2018). In this study, six genes (*VAM7*, *VPS33*, *VPS51*, *SEC22*, *GOS1* and *VPS45*) encoding proteins belong to the SNARE complex were identified sensitive to zinc stress, indicating their crucial role in the process of autophagy under excess zinc conditions. Based on these analyses, we hypothesized that autophagy may play a significant role in maintaining the cellular zinc homeostasis possibly by transporting the excess zinc to vacuolar (Figure 5).

Under the high zinc treatment, we observed 27 mutants increased the intracellular ROS levels comparing with the wild-types, consistent with their growth defect. We pick up 11 mutants for *ARO7*, *NRK1*, *SPC72*, *DBF2*, *CUE1*, *VPH1*, *LEM3*, *PLC1*, *ATG22*, *ARO1*, and *YCL007C*, which accumulated the highest relative ROS levels than that of wild-type cells in response to high zinc (Figure 3), to investigate the mechanism of oxidative damage induced by zin stress. Apparently, the expression of some antioxidant defenses genes was down-regulated by zinc stress in these mutants, suggesting that these zinc-sensitive genes might involve in maintaining the redox balance in response to high zinc. However, the rest 81 mutants accumulated similar or even lower ROS levels compared wild-type cells. Since the ROS stress was induced when the zinc concentration reached 10 mM in the wide-type cells (Pagani *et al.* 2007), these zinc-sensitive genes might be involved in ROS process when the extracellular zinc content was higher than 3 mM. They could also play a role in detoxification of excess zinc by yeast cells, but further investigations are required.

## Conclusions

In conclusion, our work has identified 108 yeast single-gene deletion mutants that are sensitive to 6 mM ZnCl_2_ by screening the yeast diploid nonessential gene deletion library. We have shown that 64 mutants accumulated higher intracellular zinc levels than wild-type cells in response to excess zinc, indicating their crucial role in maintaining the intracellular zinc homeostasis. Our data showed that the intracellular ROS levels in 51 mutants were significantly higher than that of the wild-type cells under high zinc stress. Our findings make it likely that excess zinc can generate oxidative damage to yeast cells through up-regulating several antioxidant defenses genes. Our current findings would provide a basis to understand molecular mechanisms of zinc toxicity in yeast cells.
